# Screening opportunity gap in COPD evaluation after low-dose CT findings: implications for risk-based spirometry in primary care

**DOI:** 10.1186/s12890-026-04549-9

**Published:** 2026-08-01

**Authors:** Takamasa Hotta, Mika Nakao, Shintaro Shimizu, Kashu Kitani, Ken Yoshihara, Mika Horie, Kento Kono, Seiko Tanaka, Yoshihiro Amano, Megumi Hamaguchi, Tamio Okimoto

**Affiliations:** Department of Internal Medicine, Division of Medical Oncology & Respiratory Medicine, Shimane University Faculty of Medicine, 89-1 Enya-cho, Izumo, Shimane, 693-8501 Japan

**Keywords:** Chronic obstructive pulmonary disease, Low-dose CT screening, Spirometry, Airflow limitation, Incidental findings, Lung cancer screening

## Abstract

**Background:**

Low-dose chest computed tomography (CT) frequently detects findings suggestive of chronic obstructive pulmonary disease (COPD). However, the pathway from CT detection to physiological confirmation remains poorly characterized in real-world clinical practice. This study evaluated follow-up pathways after CT findings suggestive of COPD and assessed whether simple clinical factors could identify individuals at high risk of airflow limitation.

**Methods:**

This retrospective observational study analyzed data from a voluntary, self-paid, community-based low-dose chest CT screening program in Japan that was open regardless of age or smoking history. Among 34,425 examinations conducted from fiscal year 2015 through February 2025, 1,336 examinations referred for suspected COPD were analyzed. Follow-up pathways were categorized as no documented follow-up, follow-up without spirometry, and follow-up with spirometry. Two multivariable logistic regression models evaluated documented follow-up and spirometry use. Airflow limitation was defined as a post-bronchodilator FEV₁/FVC ratio < 0.70.

**Results:**

Among 1,336 examinations, 305 (22.8%) had no documented follow-up, 556 (41.6%) had documented follow-up without spirometry, and 475 (35.6%) had documented follow-up with spirometry. Among those who underwent spirometry, airflow limitation was identified in 298 (62.7%). Current smoking was associated with lower documented follow-up, whereas older age and greater cumulative smoking exposure were associated with spirometry use. Concurrent suspicion of lung cancer was associated with a lower likelihood of spirometry use. Airflow limitation rates increased according to simple clinical risk factors. Examinations with all three risk factors—age ≥ 70 years, pack-years ≥ 40, and respiratory symptoms—had an airflow limitation prevalence of 79.8%. Notably, 90 examinations without spirometry met these high-risk criteria.

**Conclusion:**

In this community-based CT screening program, substantial gaps were observed in documented follow-up after referral for suspected COPD and subsequent spirometry use despite the high prevalence of airflow limitation among those tested. Simple clinical factors may help identify high-risk individuals and improve targeted spirometry use in routine clinical practice.

**Supplementary Information:**

The online version contains supplementary material available at https://doi.org/10.1186/s12890-026-04549-9.

## Background

Low-dose chest computed tomography (CT) screening has been widely implemented for the early detection of lung cancer and has been shown to reduce lung cancer–specific mortality in large randomized trials [1, 2]. As CT screening programs have expanded worldwide, increasing attention has been directed toward the detection of thoracic abnormalities beyond lung cancer.

Chronic obstructive pulmonary disease (COPD) is a major cause of morbidity and mortality worldwide; however, a substantial proportion of cases remain undiagnosed [3]. Spirometry is the standard diagnostic test for confirming airflow limitation; however, it remains underutilized in routine clinical practice [4]. Low-dose CT may identify emphysematous or other structural abnormalities suggestive of smoking-related lung injury, including in individuals without a previous diagnosis of COPD. However, CT findings alone do not establish the presence of airflow limitation, and spirometry remains necessary for diagnostic confirmation [5].

However, the clinical pathway following CT findings suggestive of COPD in screening settings remains unclear. Although CT screening programs are primarily designed to detect lung cancer, incidental findings related to COPD may require additional evaluation to establish a physiological diagnosis [6, 7]. In real-world clinical practice, it remains uncertain how frequently individuals with CT findings suggestive of COPD undergo clinical evaluation and spirometry after referral. Furthermore, when multiple abnormalities are detected on the same CT scan, clinical attention may be directed primarily toward the evaluation of suspected lung cancer, potentially influencing the diagnostic evaluation of COPD.

Among individuals referred for suspected COPD, simple clinical characteristics such as age, cumulative smoking exposure, and respiratory symptoms may help identify those with a higher prevalence of airflow limitation [8]. However, whether CT-based referral provides incremental predictive value beyond these clinical factors remains uncertain.

In this study, we analyzed the follow-up pathways after CT findings suggestive of COPD in a large community-based low-dose CT screening program. Specifically, we aimed to (1) describe the patterns of documented follow-up after referral for suspected COPD, (2) identify factors associated with documented follow-up and subsequent spirometry use, (3) examine the prevalence of airflow limitation among examinations with spirometry, and (4) exploratorily describe airflow limitation according to simple clinical risk factors and identify high-risk examinations without spirometry. By characterizing the pathway from CT findings to spirometric assessment, this study sought to identify potential gaps within the post-referral clinical pathway.

## Methods

### Study design and setting

This retrospective observational study was conducted using data from the JA Shimane Koseiren low-dose chest CT screening program, a community-based screening initiative conducted in collaboration with Shimane University Hospital. JA Shimane Koseiren is a prefectural health and welfare organization within the Japan Agricultural Cooperatives group that provides community-based and mobile health examinations for agricultural cooperative members, employees, and local residents in collaboration with regional JA offices, municipalities, health insurers, and workplaces. During the study period, organized population-based lung cancer screening in Japan was primarily based on chest radiography, with sputum cytology additionally offered to selected high-risk individuals. The LDCT program evaluated in this study was conducted independently of this nationally standardized screening framework.

Information about the program was distributed through health-check announcements issued by JA Shimane Koseiren through its community health-check network, and interested individuals enrolled voluntarily. No specific eligibility restrictions based on age or smoking history were applied, and both never-smokers and individuals with current or previous smoking histories could participate. Screening costs were paid out of pocket by the participants.

The program provides annual voluntary low-dose CT examinations for the early detection of lung cancer and other thoracic diseases. Although the program was conducted primarily as an LDCT lung cancer screening program, clinically relevant findings suggestive of COPD or emphysema, interstitial lung abnormalities or interstitial lung disease, and nontuberculous mycobacterial pulmonary disease were also assessed. Participants were informed before screening that LDCT could identify thoracic diseases other than lung cancer and that further clinical evaluation might be recommended.

Routine repeat screening was offered annually. The mobile CT unit visited participating municipalities according to a fixed yearly schedule, with each municipality generally screened during the same period each year. Repeat examinations were therefore usually performed at approximately 12-month intervals, although no formal minimum interval was specified.

The present analysis was restricted to fiscal year 2015 through February 2025 because spirometry-related follow-up information was not systematically collected before FY2015 and explicit institutional referral criteria for suspected COPD were introduced in FY2015. The same mobile CT unit and image-acquisition protocol were used throughout the study period. This study was conducted in accordance with the Strengthening the Reporting of Observational Studies in Epidemiology (STROBE) guidelines.

### Study population

Among 34,425 LDCT screening examinations performed from fiscal year 2015 through February 2025, 3,934 examinations had COPD-related abnormalities on screening CT. Of these, 1,346 met the institutional referral criteria for suspected COPD and were referred for further evaluation, whereas 2,588 did not meet these criteria. Across all disease categories, 2,883 examinations were judged to require further evaluation for at least one thoracic abnormality. The principal referral indications were suspected lung cancer (*n* = 1,007), suspected COPD (*n* = 1,346), interstitial lung abnormalities or interstitial lung disease (*n* = 374), and nontuberculous mycobacterial pulmonary disease (*n* = 195). These categories were not mutually exclusive, and a single examination could have more than one referral indication. Individuals with previously diagnosed respiratory diseases, including COPD, were not automatically excluded.

**Fig. 1:**
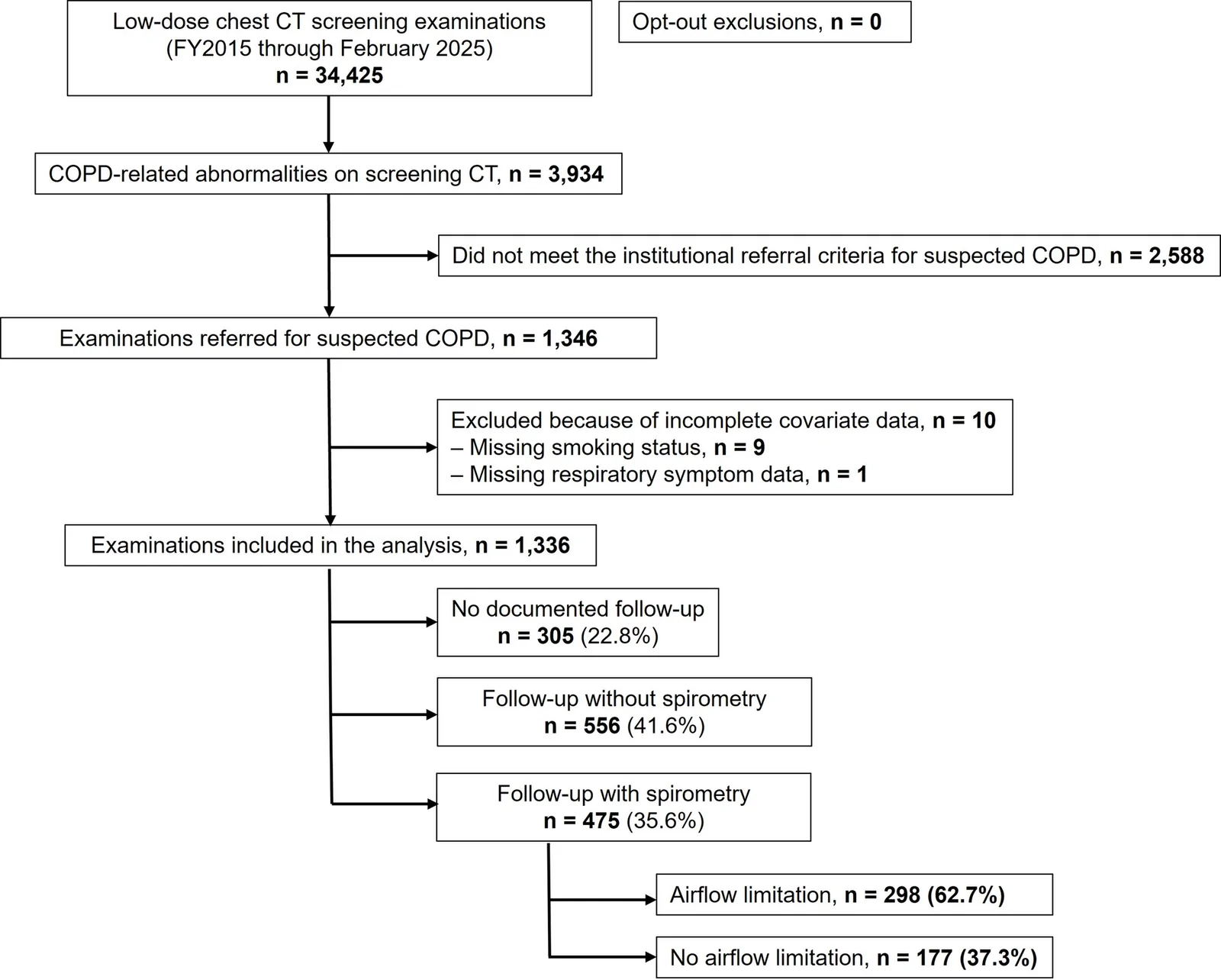
Study flow diagram of follow-up after CT findings suggestive of COPD in a community-based low-dose chest CT screening program. Among 34,425 low-dose chest CT screening examinations, 3,934 had COPD-related abnormalities; 1,346 met the institutional referral criteria for suspected COPD, whereas 2,588 did not. After excluding 10 examinations with incomplete covariate data, 1,336 examinations were included in the analysis. Counts represent screening examinations rather than unique participants. Abbreviations: COPD, chronic obstructive pulmonary disease; CT, computed tomography; FY, fiscal year

Of the 1,346 examinations referred for further evaluation of suspected COPD, 10 examinations with incomplete covariate data were excluded, leaving 1,336 examinations for the final analysis (Fig. 1).

Each screening examination was treated as an independent observation. Repeated eligible examinations from the same participant could therefore be included in the primary analysis.

### CT interpretation and referral criteria

All LDCT examinations underwent independent double reading by two board-certified respiratory physicians. Additional certification in CT interpretation was not formally required for participation in the program; however, as an institutional quality-assurance measure, at least one of the two readers was certified in lung cancer CT screening. Discrepant interpretations of pulmonary findings were jointly reviewed, and the final classification and referral decision were determined by consensus. Radiologists were not involved in resolving pulmonary interpretation discrepancies.

Referrals for further clinical evaluation were determined based on predefined institutional criteria integrating radiological findings, clinical symptoms, smoking history, and longitudinal imaging findings. The criteria covered suspected lung cancer, COPD, interstitial lung abnormalities or interstitial lung disease, and nontuberculous mycobacterial pulmonary disease and are detailed in Supplementary Table 1. The referral criteria for suspected COPD remained unchanged throughout the study period. The pulmonary nodule size threshold for referral was 5 mm during FY2015–FY2016 and was revised to 6 mm from FY2017 in accordance with updated Japanese CT screening guidelines.

For clinically important incidental findings outside respiratory medicine, advice from the relevant specialist departments was incorporated during the program’s annual quality-control review to establish and disseminate shared reporting and referral criteria.

### Classification of follow-up pathways

When further evaluation was recommended, the referral letter included a response section to be completed by the receiving medical institution and returned to the screening program. Participants were instructed to present the referral letter at the follow-up visit. An accompanying information sheet listed medical institutions with respiratory specialists that were available to accept referrals, and participants could select a follow-up facility from this list. The returned documentation recorded attendance, examinations performed, and the subsequent clinical assessment or management plan.

Follow-up pathways after referral for suspected COPD were categorized into three groups based on the referral response returned within 365 days after the screening examination: (1) no documented follow-up, defined as no returned documentation of clinical evaluation within 365 days; (2) follow-up without spirometry, defined as documented clinical evaluation without spirometry; and (3) follow-up with spirometry, defined as documented clinical evaluation with spirometry. Examinations for which no response was received within this period were classified as having no documented follow-up.

These categories reflect real-world clinical management following CT findings suggestive of COPD.

### Spirometry and definition of airflow limitation

Among examinations in which spirometry was performed, airflow limitation was defined as a post-bronchodilator FEV₁/FVC ratio < 0.70. For descriptive assessment of the severity of airflow obstruction, pre-bronchodilator FEV₁ percent predicted was summarized when available. GOLD spirometric grades were not assigned because post-bronchodilator FEV₁ percent predicted was not systematically available.

### Covariates and questionnaire data

At each screening examination, participants completed a standardized questionnaire. Routinely collected information included age, sex, height, weight, smoking status, cumulative smoking exposure, exposure to secondhand smoke, respiratory symptoms, history of respiratory diseases, diseases currently under treatment, and occupational or environmental dust exposure. Questionnaire information was collected at each screening examination but was not routinely updated between screening visits.

The prespecified covariates included age, sex, current smoking, cumulative smoking exposure (pack-years), respiratory symptoms, previous COPD diagnosis, concurrent suspicion of lung cancer on screening CT, and screening year. Respiratory symptoms included cough, sputum production, dyspnea, wheezing, and other respiratory complaints experienced during the preceding six months. Smoking status was categorized as current smoking, former smoking, or never smoking for descriptive analyses. For the multivariable analyses, current smoking was entered as a binary variable. Screening year was centered at FY2015 and entered as a continuous variable.

### Statistical analysis

Baseline characteristics across the three follow-up pathways were compared using the Kruskal–Wallis test for continuous variables and the χ² test for categorical variables. Among examinations with spirometry, characteristics according to airflow limitation status were compared using the Mann–Whitney U test for continuous variables and the χ² test for categorical variables.

Two multivariable binary logistic regression models were constructed to reflect the sequential clinical pathway. Model 1 evaluated factors associated with documented follow-up versus no documented follow-up among all examinations included in the analysis. Model 2 evaluated factors associated with spirometry use versus no spirometry among examinations with documented follow-up. Both models included age per 10-year increase, male sex, current smoking, cumulative smoking exposure per 10-pack-year increase, respiratory symptoms, previous COPD diagnosis, concurrent suspicion of lung cancer, and screening year per 1-year increase. Screening year was centered at FY2015. Adjusted odds ratios (ORs) and 95% confidence intervals (CIs) were calculated.

Missing data were handled using a complete-case approach. Examinations with missing values for any prespecified covariate were excluded before analysis, and no imputation was performed.

Exploratory analyses examined the prevalence of airflow limitation according to three simple clinical risk factors: age ≥ 70 years, cumulative smoking exposure ≥ 40 pack-years, and respiratory symptoms. The frequency of examinations meeting all three criteria among those without spirometry was also summarized. These analyses were exploratory and were performed without statistical testing.

Two sensitivity analyses were performed. First, because stable pseudonymous participant identifiers were available only from FY2021 onward, both multivariable models were repeated using only the earliest eligible examination per participant during FY2021–FY2024. Second, to address the revision of the pulmonary nodule referral threshold from 5 mm to 6 mm in FY2017, both models were repeated after restricting the cohort to examinations performed from FY2017 onward. The same covariates as in the primary analyses were used in both sensitivity analyses.

All statistical tests were two-sided, and *P*<.05 was considered statistically significant. No adjustment for multiple testing was performed. The two multivariable models were regarded as the principal analyses, whereas P values from baseline comparisons and secondary analyses were unadjusted and should be interpreted cautiously. Analyses were performed using SPSS Statistics (version 27; IBM Corp., Armonk, NY, USA) and GraphPad Prism (version 9; GraphPad Software).

### Ethical considerations

This study was approved by the Institutional Review Board of Shimane University Faculty of Medicine (Approval No. 8872; November 20, 2025). The requirement for written informed consent was waived because of the retrospective observational design. Study information was publicly disclosed through an opt-out notice posted on the website of Shimane University Faculty of Medicine. The notice described the study purpose, the screening data to be used, contact information, and the procedure for declining participation. The notice served as a publicly accessible means of providing study information and an opportunity to decline participation. No participant opted out of the use of their data for this study.

The original screening records, including personally identifiable information, were managed by JA Shimane Koseiren. Before transfer to the research team, all direct personal identifiers were removed, and the research team received only de-identified data. Beginning in FY2021, JA Shimane Koseiren assigned each participant a stable pseudonymous identifier that enabled repeated screening examinations from the same individual to be linked without disclosing the participant’s identity. Stable participant-level identifiers were unavailable before FY2021.

JA Shimane Koseiren retained the identifiable source records and the linkage information. If a participant had opted out, the corresponding records would have been identified and excluded by JA Shimane Koseiren before de-identification and transfer to the research team. The research team had no access to names, addresses, or other directly identifying information.

All procedures were conducted in accordance with the Declaration of Helsinki and its amendments. This manuscript was prepared in accordance with the STROBE reporting guideline for observational studies.

## Results

### Study population

**Fig. 2:**
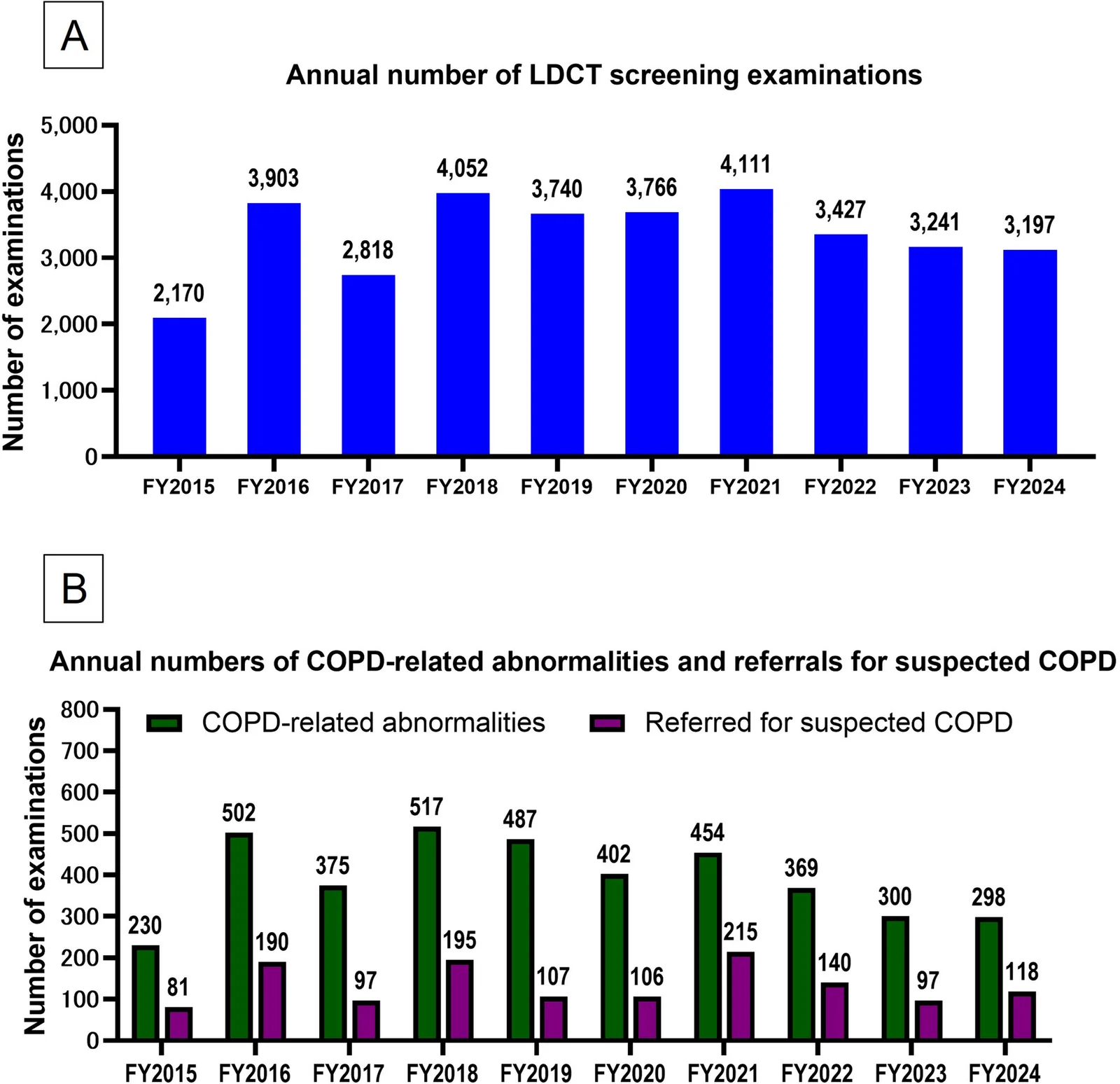
Annual volume of low-dose chest CT screening examinations and COPD-related findings during the study period. **A** Annual number of low-dose chest CT screening examinations. **B **Annual numbers of examinations with COPD-related abnormalities and examinations referred for further evaluation of suspected COPD. Counts represent screening examinations rather than unique participants. FY2024 includes examinations performed through February 2025. Abbreviations: COPD, chronic obstructive pulmonary disease; FY, fiscal year; LDCT, low-dose computed tomography

Among 34,425 LDCT screening examinations performed from FY2015 through February 2025, 1,346 examinations resulted in referral for further evaluation of suspected COPD. After excluding 10 examinations with incomplete covariate data, 1,336 examinations were included in the final analysis (Fig. 1). Among all 34,425 LDCT screening examinations, 17,867 (51.9%) involved male participants, whereas 1,284 of the 1,336 examinations included in the final analysis (96.1%) involved male participants. Of these, 305 (22.8%) had no documented follow-up, whereas 1,031 (77.2%) had documented follow-up within 365 days. Overall, 556 examinations (41.6%) had documented follow-up without spirometry, and 475 (35.6%) had documented follow-up with spirometry. Among the 475 examinations with spirometry, airflow limitation was identified in 298 (62.7%), whereas 177 (37.3%) showed no airflow limitation. The annual numbers of LDCT screening examinations, COPD-related abnormalities, and referrals for suspected COPD are shown in Fig. 2. Annual screening volume ranged from 2,170 to 4,111 examinations, and the annual number of referrals for suspected COPD ranged from 81 to 215 examinations during the study period.

### Baseline characteristics according to follow-up pathway

Baseline characteristics according to the follow-up pathway are presented in Table 1. Age, current smoking, cumulative smoking exposure, respiratory symptoms, and concurrent suspicion of lung cancer differed significantly across the three follow-up pathways. The median age was 67 years in both the no documented follow-up and follow-up without spirometry groups and 69 years in the follow-up with spirometry group (*P*<.001).

Current smoking was most frequent in the no documented follow-up group (80.7%), compared with 62.6% in the follow-up without spirometry group and 59.4% in the follow-up with spirometry group (*P*<.001). Median cumulative smoking exposure was highest in the follow-up with spirometry group (44.0 pack-years), compared with 40.0 and 39.0 pack-years in the no documented follow-up and follow-up without spirometry groups, respectively (*P*=.001).

Respiratory symptoms were present in 44.3%, 48.4%, and 53.7% of examinations in the no documented follow-up, follow-up without spirometry, and follow-up with spirometry groups, respectively (*P*=.031). Concurrent suspicion of lung cancer was most frequent in the follow-up without spirometry group (22.7%), compared with 10.5% in the no documented follow-up group and 7.6% in the follow-up with spirometry group (*P*<.001). Male sex and previous COPD diagnosis did not differ significantly across the pathways.

**Table 1:** Baseline characteristics according to follow-up pathway after referral for suspected COPD

Variable	No documented follow-up (*n* = 305)	Follow-up without spirometry (*n* = 556)	Follow-up with spirometry (*n* = 475)	*P* value
Age, years, median (IQR) ^a^	67 (59–72)	67 (62–73)	69 (64–74)	< 0.001
Male sex, n (%) ^b^	294 (96.4)	538 (96.8)	452 (95.2)	0.397
Current smoking, n (%) ^b^	246 (80.7)	348 (62.6)	282 (59.4)	< 0.001
Pack-years, median (IQR) ^a^	40.0 (30.0–51.0)	39.0 (27.5–50.0)	44.0 (31.0–54.0)	0.001
Respiratory symptoms, n (%) ^b^	135 (44.3)	269 (48.4)	255 (53.7)	0.031
Previous COPD diagnosis, n (%) ^b^	38 (12.5)	67 (12.1)	65 (13.7)	0.726
Concurrent suspicion of lung cancer, n (%)^b^	32 (10.5)	126 (22.7)	36 (7.6)	< 0.001

Values are presented as median (interquartile range [IQR]) or number (percentage). Non-current smoking included former and never smoking. Respiratory symptoms included cough, sputum production, dyspnea, wheezing, or other respiratory complaints reported within the preceding six months

*Abbreviations*: *COPD *chronic obstructive pulmonary disease, *IQR* interquartile range

ᵃ Kruskal–Wallis test

ᵇ χ² test

### Characteristics of airflow limitation

Characteristics of examinations with and without airflow limitation among those who underwent spirometry are shown in Table 2. Examinations with airflow limitation were older than those without airflow limitation, with median ages of 71 and 67 years, respectively (*P*<.001). Cumulative smoking exposure was also greater in examinations with airflow limitation (median, 45.0 vs. 42.0 pack-years; *P*=.019).

A previous COPD diagnosis was more frequent among examinations with airflow limitation than among those without airflow limitation (16.8% vs. 8.5%; *P*=.011). Male sex, current smoking, respiratory symptoms, and concurrent suspicion of lung cancer did not differ significantly between the two groups.

Among the 298 examinations with airflow limitation, pre-bronchodilator FEV₁ percent predicted was available for 289 (97.0%). The median pre-bronchodilator FEV₁ was 78.6% predicted (IQR, 65.7%–91.0%). GOLD spirometric grades were not assigned because post-bronchodilator FEV₁ percent predicted was not systematically available.

**Table 2:** Characteristics of examinations with and without airflow limitation among those who underwent spirometry

Variable	Airflow limitation (*n* = 298)	No airflow limitation (*n* = 177)	*P* value
Age, years, median (IQR) ^a^	71 (66–74)	67 (59–71.5)	< 0.001
Male sex, n (%) ᵇ	281 (94.3)	171 (96.6)	0.256
Current smoking, n (%) ᵇ	177 (59.4)	105 (59.3)	0.987
Pack-years, median (IQR) ^a^	45.0 (33.0–56.4)	42.0 (30.0–49.5)	0.019
Respiratory symptoms, n (%) ᵇ	168 (56.4)	87 (49.2)	0.127
Previous COPD diagnosis, n (%) ᵇ	50 (16.8)	15 (8.5)	0.011
Concurrent suspicion of lung cancer, n (%) ᵇ	23 (7.7)	13 (7.3)	0.882

Values are presented as median (interquartile range [IQR]) or number (percentage). Non-current smoking included former and never smoking. Respiratory symptoms included cough, sputum production, dyspnea, wheezing, or other respiratory complaints reported within the preceding six months. Airflow limitation was defined as a post-bronchodilator FEV₁/FVC ratio < 0.70

*Abbreviations*: *COPD *chronic obstructive pulmonary disease,* FEV₁ *forced expiratory volume in one second, *FVC *forced vital capacity, *IQR *interquartile range

ᵃ Mann–Whitney U test

ᵇ χ² test

### Factors associated with documented follow-up and spirometry use

The results of the two multivariable logistic regression models are shown in Table 3. In Model 1, current smoking was associated with lower odds of documented follow-up (adjusted OR, 0.38; 95% CI, 0.27–0.52; *P*<.001), whereas respiratory symptoms were associated with higher odds of documented follow-up (adjusted OR, 1.36; 95% CI, 1.04–1.78; *P*=.023). Later screening year was also associated with lower odds of documented follow-up (adjusted OR per 1-year increase, 0.90; 95% CI, 0.86–0.95; *P*<.001).

In Model 2, among examinations with documented follow-up, older age (adjusted OR per 10-year increase, 1.26; 95% CI, 1.08–1.47; *P*=.003), greater cumulative smoking exposure (adjusted OR per 10-pack-year increase, 1.13; 95% CI, 1.06–1.20; *P*<.001), and later screening year (adjusted OR per 1-year increase, 1.12; 95% CI, 1.07–1.18; *P*<.001) were associated with higher odds of spirometry use. Male sex (adjusted OR, 0.49; 95% CI, 0.25–0.96; *P*=.037) and concurrent suspicion of lung cancer (adjusted OR, 0.25; 95% CI, 0.16–0.37; *P*<.001) were associated with lower odds of spirometry use.

During FY2021–FY2024, 563 eligible examinations corresponded to 517 unique participants. Of these, 38 participants contributed more than one eligible examination, accounting for 84 examinations in total. Thus, 46 examinations were repeat examinations beyond the earliest eligible examination for each participant, representing 8.2% of the eligible examinations during this period.

In the sensitivity analysis restricted to the earliest eligible examination per participant during FY2021–FY2024, concurrent suspicion of lung cancer remained associated with lower spirometry use (adjusted OR, 0.32; 95% CI, 0.17–0.58; *P*<.001). In the documented follow-up model, the directions of the associations for current smoking, concurrent suspicion of lung cancer, and screening year were consistent with those in the primary analysis, although they did not reach statistical significance in the smaller participant-level cohort (Supplementary Table 2).

In analyses restricted to examinations performed from FY2017 onward, current smoking remained associated with lower documented follow-up (adjusted OR, 0.37; 95% CI, 0.26–0.53; *P*<.001). Older age and greater cumulative smoking exposure remained associated with higher spirometry use, whereas concurrent suspicion of lung cancer remained strongly associated with lower spirometry use (adjusted OR, 0.25; 95% CI, 0.16–0.39; *P*<.001) (Supplementary Table 3).

**Table 3:** Multivariable logistic regression analyses of documented follow-up and spirometry use after referral for suspected COPD

Variable	Model 1: Documented follow-up, adjusted OR (95% CI)	*P* value	Model 2: Spirometry use, adjusted OR (95% CI)	*P* value
Age, per 10-year increase	1.14 (0.98–1.33)	0.083	1.26(1.08–1.47)	0.003
Male sex	0.81(0.40–1.64)	0.561	0.49(0.25–0.96)	0.037
Current smoking	0.38(0.27–0.52)	< 0.001	0.98(0.73–1.30)	0.877
Pack-years, per 10	0.98(0.91–1.05)	0.526	1.13(1.06–1.20)	< 0.001
Respiratory symptoms	1.36(1.04–1.78)	0.023	1.17(0.90–1.52)	0.243
Previous COPD diagnosis	0.73(0.48–1.10)	0.128	1.10(0.74–1.64)	0.636
Concurrent suspicion of lung cancer	1.46(0.96–2.20)	0.077	0.25(0.16–0.37)	< 0.001
Screening year, per 1-year increase	0.90(0.86–0.95)	< 0.001	1.12(1.07–1.18)	< 0.001

Model 1 included all 1,336 examinations and evaluated documented follow-up versus no documented follow-up. Model 2 included the 1,031 examinations with documented follow-up and evaluated follow-up with spirometry versus follow-up without spirometry. Both models simultaneously included all variables listed in the table. Reference categories were female sex, non-current smoking, absence of respiratory symptoms, no previous COPD diagnosis, and no concurrent suspicion of lung cancer. Screening year was centered at 2015 and entered as a continuous variable. Adjusted ORs greater than 1 indicate higher odds of the respective outcome. Non-current smoking included former and never smoking

*Abbreviations*: *CI *confidence interval, *COPD *chronic obstructive pulmonary disease, *OR *odds ratio

### Exploratory airflow limitation rates according to simple clinical risk factors

In exploratory analyses performed without statistical testing, airflow limitation rates according to simple clinical risk factors are shown in Table 4. Among examinations with spirometry, airflow limitation was observed in 52.2% of those aged < 70 years and 73.9% of those aged ≥ 70 years. The corresponding rates were 59.3% and 64.8% for cumulative smoking exposure < 40 and ≥ 40 pack-years, respectively, and 59.1% and 65.9% for examinations without and with respiratory symptoms, respectively.

Airflow limitation was observed in 71 of 89 examinations (79.8%) with all three risk factors: age ≥ 70 years, cumulative smoking exposure ≥ 40 pack-years, and respiratory symptoms. Among the 861 examinations without spirometry, 90 (10.5%) met all three high-risk criteria, including 27 of 305 examinations (8.9%) with no documented follow-up and 63 of 556 examinations (11.3%) with documented follow-up without spirometry.

**Table 4:** Exploratory airflow limitation rates according to simple clinical risk factors and high-risk examinations without spirometry

Panel A. Airflow limitation rates among examinations with spirometry		
Risk factor pattern	*n*	Airflow limitation, *n* (%)
Overall	475	298 (62.7)
Age < 70 years	245	128 (52.2)
Age ≥ 70 years	230	170 (73.9)
Pack-years < 40	182	108 (59.3)
Pack-years ≥ 40	293	190 (64.8)
No respiratory symptoms	220	130 (59.1)
Respiratory symptoms present	255	168 (65.9)
Age ≥ 70 years + pack-years ≥ 40	144	109 (75.7)
Age ≥ 70 years + respiratory symptoms	128	98 (76.6)
Pack-years ≥ 40 + respiratory symptoms	167	118 (70.7)
Age ≥ 70 years + pack-years ≥ 40 + respiratory symptoms	89	71 (79.8)

Panel B. High-risk examinations without spirometry		
Follow-up pathway	**n**	**High-risk examinations**, * n (%)
No documented follow-up	305	27 (8.9)
Documented follow-up without spirometry	556	63 (11.3)
Overall without spirometry	861	90 (10.5)

Values are presented as number (percentage). Respiratory symptoms included cough, sputum production, dyspnea, wheezing, or other respiratory complaints reported within the preceding six months. Airflow limitation was defined as a post-bronchodilator FEV₁/FVC ratio < 0.70. This analysis was exploratory, and no statistical testing was performed. Percentages in Panel A were calculated within each risk-factor pattern, and percentages in Panel B were calculated within each follow-up pathway. Airflow limitation status was unavailable for examinations without spirometry

*Abbreviations*: *FEV₁ *forced expiratory volume in one second, *FVC *forced vital capacity

*High risk was defined as the presence of all three factors: age ≥ 70 years, cumulative smoking exposure ≥ 40 pack-years, and respiratory symptoms

## Discussion

In this retrospective study of a community-based low-dose chest CT screening program, we characterized the clinical pathway after referral for suspected COPD. Among 1,336 examinations, 22.8% had no documented follow-up, and spirometry was performed in 35.6%. Among examinations with spirometry, airflow limitation was identified in 62.7%. Current smoking was associated with lower odds of documented follow-up, whereas respiratory symptoms were associated with higher odds of documented follow-up. Among examinations with documented follow-up, older age and greater cumulative smoking exposure were associated with higher odds of spirometry use, whereas male sex and concurrent suspicion of lung cancer were associated with lower odds of spirometry use.

The marked male predominance among examinations referred for suspected COPD did not reflect the sex distribution of the overall screening examinations (96.1% vs. 51.9%). In Japan, current habitual smoking is substantially more prevalent among older men than women [9]. Because smoking history was incorporated into the institutional referral criteria for suspected COPD, this sex difference may partly explain the predominance of men in the referred cohort, although other unmeasured factors may also have contributed.

These findings identify gaps within the clinical pathway after COPD-related CT findings had already prompted referral. LDCT may reveal emphysematous or other structural abnormalities suggestive of COPD, including in individuals without a previous diagnosis; however, such findings alone do not establish airflow limitation, and spirometry remains necessary for physiological confirmation [5, 6]. Accordingly, the present results should not be interpreted as demonstrating early COPD detection by LDCT itself. Rather, they show that a substantial proportion of examinations referred for suspected COPD did not proceed to documented spirometric assessment.

The association between current smoking and lower documented follow-up suggests that current smokers may be less likely to complete, or have documentation of, post-referral evaluation. In contrast, respiratory symptoms were associated with documented follow-up but not with spirometry use among those who attended. This distinction indicates that factors influencing attendance after referral may differ from those influencing the decision to perform spirometry after attendance. Interventions to improve the pathway may therefore need to address these two stages separately.

Concurrent suspicion of lung cancer was strongly associated with lower spirometry use among examinations with documented follow-up. This finding may reflect diagnostic competition between evaluation of a potentially malignant lesion and assessment of suspected COPD. However, it should not necessarily be interpreted as clinical oversight. When lung cancer is suspected, clinicians may appropriately prioritize diagnostic imaging, bronchoscopy, tissue sampling, or surgical evaluation, and spirometry may be intentionally deferred during this higher-priority workup. Conversely, COPD evaluation may not subsequently be revisited after completion of the malignancy assessment. Because our database did not capture the timing of spirometry relative to the lung cancer workup or the reasons for deferral, intentional postponement could not be distinguished from failure to resume COPD evaluation.

The exploratory analysis suggested that simple clinical characteristics may help identify examinations with a high prevalence of airflow limitation. Airflow limitation was present in 79.8% of examinations with all three factors—age ≥ 70 years, cumulative smoking exposure ≥ 40 pack-years, and respiratory symptoms—while 90 examinations without spirometry met these criteria. These findings may support prioritization of spirometry after referral when clinical resources are limited. Nevertheless, this analysis was exploratory and was performed without statistical testing.

Temporal changes were also considered. The same mobile CT unit, image-acquisition protocol, and COPD referral criteria were used throughout the study period. However, the pulmonary nodule referral threshold changed from 5 mm to 6 mm in FY2017, which may have affected the composition of examinations with concurrent suspicion of lung cancer. Screening year was therefore included in both multivariable models, and sensitivity analyses were restricted to examinations performed from FY2017 onward. The associations of current smoking with lower documented follow-up and of concurrent suspicion of lung cancer with lower spirometry use were maintained. In addition, the inverse association between concurrent suspicion of lung cancer and spirometry use remained significant in the participant-level sensitivity analysis restricted to the earliest eligible examination during FY2021–FY2024. These findings support the robustness of the principal observation concerning concurrent lung cancer suspicion, although several other associations were attenuated in the smaller participant-level cohort.

The present findings should not be interpreted as establishing that systematic COPD screening should routinely be incorporated into all lung cancer screening programs. LDCT screening requires substantial resources, and broadening its scope to multiple diseases could divert attention and clinical resources from its primary purpose of lung cancer detection. Moreover, identifying additional structural abnormalities does not necessarily translate into patient benefit unless subsequent diagnosis and treatment improve clinically meaningful outcomes. Our study focused on individuals for whom a decision to recommend further evaluation for suspected COPD had already been made. Thus, the observed underuse of spirometry represents a gap within an existing post-referral pathway rather than evidence that LDCT should be used primarily as a COPD screening modality. Future prospective studies should evaluate diagnostic yield, treatment initiation, respiratory symptoms, quality of life, survival, potential harms, competing clinical priorities, and resource use before the net benefit of integrating COPD assessment into lung cancer screening can be determined.

This study had several limitations. First, the screening examination rather than the individual participant was used as the unit of analysis, and repeated eligible examinations from the same participant could have been included. Stable pseudonymous participant identifiers were unavailable before FY2021; therefore, within-participant correlation could not be accounted for over the entire study period. However, a sensitivity analysis restricted to the earliest eligible examination per participant during FY2021–FY2024 showed a similar inverse association between concurrent suspicion of lung cancer and spirometry use, although several other associations were attenuated in the smaller participant-level cohort.

Second, documented follow-up was ascertained from referral response forms returned by receiving medical institutions. Participants who attended a medical institution without presenting the referral letter, or whose response form was not returned, may therefore have been misclassified as having no documented follow-up. Consequently, the observed proportion of examinations without documented follow-up may overestimate true non-attendance.

Third, spirometry was performed at the discretion of the treating physicians. Individuals perceived to have a higher probability of airflow limitation may have been preferentially selected for testing; therefore, the observed prevalence of airflow limitation among examinations with spirometry may not represent that among all examinations referred for suspected COPD. Moreover, because spirometry was not systematically performed in participants who were not referred, we could not evaluate the sensitivity, specificity, false-positive rate, false-negative rate, or incremental predictive value of the CT-based referral criteria.

Fourth, the database did not capture the timing of spirometry relative to the diagnostic workup for suspected lung cancer or the clinical reasons for deferring spirometry. Thus, lower spirometry use in examinations with concurrent suspicion of lung cancer may have reflected either intentional postponement during a higher-priority malignancy workup or failure to resume COPD evaluation afterward.

Finally, this study was conducted within a single community-based LDCT screening program in Japan. Screening eligibility, referral procedures, and follow-up practices may differ across institutions and healthcare systems, which may limit the generalizability of the findings.

## Conclusions

In this community-based LDCT screening program, substantial gaps were observed in documented follow-up after referral for suspected COPD and in subsequent spirometry use. Current smoking was associated with lower documented follow-up, whereas concurrent suspicion of lung cancer was strongly associated with lower spirometry use.

These findings identify potential areas for improvement within the clinical pathway after COPD-related CT findings have already prompted referral. However, they do not establish the net benefit of incorporating systematic COPD assessment into lung cancer screening. Prospective studies evaluating patient-centered outcomes, treatment effects, potential harms, competing clinical priorities, and resource use are needed.

## Supplementary Information

Supplementary Material 1.

## Data Availability

The datasets generated and/or analyzed during the current study are not publicly available because they contain information obtained under agreements with JA Shimane Koseiren and are subject to institutional restrictions. Data are available from the corresponding author, Dr. Tamio Okimoto (okimoto@med.shimane-u.ac.jp), upon reasonable request and with permission from JA Shimane Koseiren and the Institutional Review Board of Shimane University Faculty of Medicine.

## Abbreviations

ANOVA: analysis of variance
CI: confidence interval
COPD: chronic obstructive pulmonary disease
CT: computed tomography
FEV₁: forced expiratory volume in one second
FVC: forced vital capacity
IQR: interquartile range
OR: odds ratio
SD: standard deviation
STROBE: Strengthening the Reporting of Observational Studies in Epidemiology
