# Supplementary material for: Screening opportunity gap in COPD evaluation after low-dose CT findings: implications for risk-based spirometry in primary care
Source: BMC Pulm Med. 2026 Aug 1;26:509. doi: 10.1186/s12890-026-04549-9 (PMC13718137; doi:10.1186/s12890-026-04549-9)
Supplement: Supplementary file 1 [file 12890_2026_4549_MOESM1_ESM.docx]

**Supplementary Information**

| **Supplementary Table 1. Radiologic and clinical referral criteria for suspected disease categories** | | | | |
| --- | --- | --- | --- | --- |
| **Disease category** | **Radiologic findings** | **Clinical criteria** | **Longitudinal imaging criteria** | **Referral decision** |
| **Suspected lung cancer** | Pulmonary nodule meeting the applicable referral size threshold* | Not required | Not required | Refer |
|  | Pulmonary nodule below the applicable referral size threshold without suspicious morphology | Not required | Stable on prior imaging | Do not refer |
|  | Pulmonary nodule of any size considered suspicious for malignancy by the interpreting physicians | Not required | Not required | Refer |
| **Suspected COPD** | Emphysematous changes on CT | Respiratory symptoms in a current or former smoker | Not required | Refer |
|  | Emphysematous changes on CT | Never-smoking history or absence of respiratory symptoms | Not required | Do not refer |
|  | Previous COPD diagnosis or prior identification of suspected COPD on screening | Continued smoking without established COPD care | Not required | Refer |
|  | Previous COPD diagnosis or prior identification of suspected COPD on screening | No current smoking or established COPD care | Not required | Do not refer |
| **Suspected ILA/ILD** | Definite honeycombing | Not required | Not required | Refer |
|  | Interstitial abnormalities | Respiratory symptoms present | Not required | Refer |
|  | Interstitial abnormalities | Respiratory symptoms absent | Radiologic progression | Refer |
|  | Interstitial abnormalities | Respiratory symptoms absent | No radiologic progression | Do not refer |
| **Suspected NTM pulmonary disease** | Nodular/bronchiectatic or tree-in-bud pattern | Sputum-related respiratory symptoms present | Not required | Refer |
|  | Nodular/bronchiectatic or tree-in-bud pattern | Sputum-related respiratory symptoms absent | Radiologic progression | Refer |
|  | Nodular/bronchiectatic or tree-in-bud pattern | Sputum-related respiratory symptoms absent | No radiologic progression | Do not refer |
| Abbreviations: COPD, chronic obstructive pulmonary disease; CT, computed tomography; ILA, interstitial lung abnormality; ILD, interstitial lung disease; NTM, nontuberculous mycobacteria.  *The pulmonary nodule size threshold for referral was 5 mm during FY2015–FY2016 and was revised to 6 mm from FY2017 in accordance with updated Japanese CT screening guidelines.  Screening examinations could be assigned to more than one suspected disease category; the categories were not mutually exclusive. Referral decisions were guided by institutional criteria integrating CT findings, respiratory symptoms, smoking history, previous COPD-related information, and longitudinal imaging findings. Final referral decisions were made by the interpreting respiratory physicians through consensus. For COPD-related abnormalities, examinations that did not meet the corresponding clinical criteria were not referred for suspected COPD. | | | | |

| **Supplementary Table 2. Multivariable logistic regression sensitivity analyses restricted to the earliest eligible examination per participant during FY2021–FY2024** | | | | |
| --- | --- | --- | --- | --- |
| **Variable** | **Model 1: Documented follow-up, adjusted OR (95% CI)** | **P value** | **Model 2: Spirometry use, adjusted OR (95% CI)** | **P value** |
| Age, per 10-year increase | 1.01 **(**0.82–1.24**)** | 0.937 | 1.08 **(**0.88–1.32**)** | 0.482 |
| Male sex | 1.48 **(**0.82–2.68**)** | 0.193 | 1.10 **(**0.59–2.05**)** | 0.766 |
| Current smoking | 0.61 **(**0.36–1.01**)** | 0.055 | 0.93 **(**0.57–1.53**)** | 0.786 |
| Pack-years, per 10-pack-year increase | 0.97 **(**0.86–1.10**)** | 0.637 | 1.03 **(**0.91–1.16**)** | 0.646 |
| Previous COPD diagnosis | 1.18 **(**0.59–2.37**)** | 0.641 | 1.14 **(**0.59–2.19**)** | 0.694 |
| Respiratory symptoms | 1.20 **(**0.79–1.82**)** | 0.387 | 0.97 **(**0.64–1.47**)** | 0.874 |
| Concurrent suspicion of lung cancer | 1.92 **(**0.99–3.74**)** | 0.055 | 0.32 **(**0.17–0.58**)** | <.001 |
| Screening year, per 1-year increase | 0.83 **(**0.68–1.01**)** | 0.059 | 0.98 **(**0.79–1.21**)** | 0.860 |
| Model 1 included 517 participants and evaluated documented follow-up versus no documented follow-up. Model 2 included the 386 participants with documented follow-up and evaluated follow-up with spirometry versus follow-up without spirometry. The sensitivity analyses were restricted to the earliest eligible complete-case examination for each participant during FY2021–FY2024, when stable pseudonymous participant identifiers were available. Both models simultaneously included all variables listed in the table. Reference categories were female sex, non-current smoking, absence of respiratory symptoms, no previous COPD diagnosis, and no concurrent suspicion of lung cancer. Screening year was centered at 2015 and entered as a continuous variable. Adjusted ORs greater than 1 indicate higher odds of the respective outcome. P values were calculated using Wald tests.  Model 1: omnibus test, P=.072; Nagelkerke R²=.041; Hosmer–Lemeshow test, P=.721. Model 2: omnibus test, P=.025; Nagelkerke R²=.059; Hosmer–Lemeshow test, P=.722.  Abbreviations: CI, confidence interval; COPD, chronic obstructive pulmonary disease; FY, fiscal year; OR, odds ratio. | | | | |

| **Supplementary Table 3. Multivariable logistic regression sensitivity analyses restricted to examinations performed from FY2017 through February 2025** | | | | |
| --- | --- | --- | --- | --- |
| **Variable** | **Model 1: Documented follow-up, adjusted OR (95% CI)** | **P value** | **Model 2: Spirometry use, adjusted OR (95% CI)** | **P value** |
| Age, per 10-year increase | 1.12 **(**0.95–1.32**)** | 0.186 | 1.22 **(**1.03–1.44**)** | 0.023 |
| Male sex | 0.80 **(**0.36–1.75**)** | 0.571 | 0.61 **(**0.28–1.32**)** | 0.206 |
| Current smoking | 0.37 **(**0.26–0.53**)** | <.001 | 0.99 **(**0.72–1.36**)** | 0.946 |
| Pack-years, per 10-pack-year increase | 1.00 **(**0.93–1.09**)** | 0.923 | 1.11 **(**1.03–1.19**)** | 0.007 |
| Respiratory symptoms | 1.29 **(**0.96–1.73**)** | 0.092 | 1.15 **(**0.86–1.53**)** | 0.359 |
| Previous COPD diagnosis | 0.81 **(**0.51–1.29**)** | 0.365 | 1.31 **(**0.84–2.04**)** | 0.233 |
| Concurrent suspicion of lung cancer | 1.40 **(**0.89–2.22**)** | 0.147 | 0.25 **(**0.16–0.39**)** | <.001 |
| Screening year, per 1-year increase | 0.87 **(**0.81–0.93**)** | <.001 | 1.03 **(**0.96–1.10**)** | 0.385 |
| Model 1 included 1,066 examinations and evaluated documented follow-up versus no documented follow-up. Model 2 included the 814 examinations with documented follow-up and evaluated follow-up with spirometry versus follow-up without spirometry. The analyses were restricted to examinations performed from FY2017 through February 2025, after the pulmonary nodule referral threshold had been revised from 5 mm to 6 mm. Both models simultaneously included all variables listed in the table. Reference categories were female sex, non-current smoking, absence of respiratory symptoms, no previous COPD diagnosis, and no concurrent suspicion of lung cancer. Screening year was centered at 2015 and entered as a continuous variable. Adjusted ORs greater than 1 indicate higher odds of the respective outcome. P values were calculated using Wald tests.  Model 1: omnibus test, P<.001; Nagelkerke R²=.087; Hosmer–Lemeshow test, P=.510. Model 2: omnibus test, P<.001; Nagelkerke R²=.096; Hosmer–Lemeshow test, P=.155.  Abbreviations: CI, confidence interval; COPD, chronic obstructive pulmonary disease; FY, fiscal year; OR, odds ratio. | | | | |
